# The psychodynamic role of the displacement defense mechanism in people with obesity and anorexia nervosa

**DOI:** 10.1192/j.eurpsy.2023.1799

**Published:** 2023-07-19

**Authors:** F. Mustač, M. Matovinović, D. Marčinko

**Affiliations:** 1 Department of Psychiatry and Psychological Medicine; 2 Department of Endocrinology, University Hospital Centre Zagreb, Zagreb, Croatia

## Abstract

**Introduction:**

Feeding plays a very important role in our lives. First of all survival, but in general it has a much wider and important social role. Through feeding, in addition to satisfying life’s needs and basic instincts, we experience a sense of satisfaction through the investment of libido. Anorexia nervosa and obesity as the two extremes of eating disorders can be considered as a disturbed experience of the satisfaction of eating, and in the background there is usually a very weak, fragile personality structure. A fragile personality structure tends to use primitive defense mechanisms, but the use of healthier, neurotic defense mechanisms in people with eating disorders should not be neglected.

**Objectives:**

To investigate the role of the psychological defense mechanism of displacement in people with obesity and anorexia nervosa.

**Methods:**

Search of contemporary professional and scientific literature in the field of psychodynamics of eating disorders.

**Results:**

Displacement is a defense mechanism in which, when faced with a problem, the problem is not solved with the initial object of aggression, but the problem is moved to another object or situation that the individual perceives as less dangerous. Thus, when faced with a stressful situation or sadness, obese people may have a need for emotional eating, which can be interpreted as displacement of the problem, which is temporarily “solved” by satisfying the basic instinct, but later the person becomes overwhelmed by internal and external shame. Equally so, a traumatic upbringing and disturbed interpersonal family dynamics, which are often present in people with anorexia nervosa, can cause anxiety drives that are displaced in the form of the need for a strong restriction of food intake.

**Conclusions:**

In people with obesity and anorexia nervosa, the use of the defense mechanism of displacement is pointed out. Through psychodynamic psychotherapy it can be very useful to recognize and interpert the use of displacement and, thus to enable reaching a neurotic and healthier level of functioning in people with the aforementioned eating disorders.

**Disclosure of Interest:**

None Declared

